# Genome-Wide Identification and Expression Analysis of the Class III Peroxidase Gene Family in Potato (*Solanum tuberosum* L.)

**DOI:** 10.3389/fgene.2020.593577

**Published:** 2020-12-03

**Authors:** Xuanshong Yang, Jiazheng Yuan, Wenbin Luo, Mingyue Qin, Jiahan Yang, Weiren Wu, Xiaofang Xie

**Affiliations:** ^1^College of Life Sciences, Fujian Agriculture & Forestry University, Fuzhou, China; ^2^Fujian Key Laboratory of Crop Breeding by Design, Fujian Agriculture & Forestry University, Fuzhou, China; ^3^Department of Biological and Forensic Sciences, Fayetteville State University, Fayetteville, NC, United States; ^4^The Crop Institute, Fujian Academy of Agricultural Sciences, Fuzhou, China

**Keywords:** potato, class III peroxidase, phylogenetic analysis, expression pattern, abiotic stress

## Abstract

Class III peroxidases (PRXs) are plant-specific enzymes and play important roles in plant growth, development and stress response. In this study, a total of 102 non-redundant *PRX* gene members (*StPRXs*) were identified in potato (*Solanum tuberosum* L.). They were divided into 9 subfamilies based on phylogenetic analysis. The members of each subfamily were found to contain similar organizations of the exon/intron structures and protein motifs. The *StPRX* genes were not equally distributed among chromosomes. There were 57 gene pairs of segmental duplication and 26 gene pairs of tandem duplication. Expression pattern analysis based on the RNA-seq data of potato from public databases indicated that *StPRX* genes were expressed differently in various tissues and responded specifically to heat, salt and drought stresses. Most of the *StPRX* genes were expressed at significantly higher levels in root than in other tissues. In addition, real-time quantitative PCR (qRT-PCR) analysis for 7 selected *StPRX* genes indicated that these genes displayed various expression levels under abiotic stresses. Our results provide valuable information for better understanding the evolution of *StPRX* gene family in potato and lay the vital foundation for further exploration of *PRX* gene function in plants.

## Introduction

As a large family of isozymes, peroxidases (POD) play important roles in the growth, development and defense processes in plants (Hiraga et al., 2001). Peroxidases are divided into two major groups, hemoglobin peroxidases and non-hemoglobin peroxidases, according to their protein structures (Hiraga et al., 2000). Exception of animal peroxidases, the hemoglobin peroxidases have been further divided into three classes based on their sequences and catalytic properties, namely, I, II and III peroxidases. Class I peroxidases are widely distributed in most living organisms other than animals, and play an important role in removing excess H_2_O_2_ to prevent cell damage (Erman and Vitello, 2002; Shigeoka et al., 2002). Class II peroxidases only present in fungi and are mainly involved in lignin degradation (Piontek et al., 2001). Class III peroxidases (PRX, EC 1.11.1.7) exist in various plants as a multi-genic family (Tognolli et al., 2002; Duroux and Welinder, 2003; Passardi et al., 2004a). The PRX protein contains highly conserved amino acid residues, including a single peptide chain and the protoporphyrin IX domain (Welinder, 1993). Most plant PRXs fuse with carbohydrate side chains to form glycosylated proteins. This glycosylation prevents the protein being degraded by protease and maintains the enzyme stability (Zheng and Van Huystee, 1991). In addition, two histidine residues interact with a heme group and eight cysteine residues, forming disulfide bridges; the distal histidine is essential for catalytic activity (Passardi et al., 2004a). The functions of PRXs have been illustrated in several studies as important proteins for a wide range of physiological processes of plants, such as growth hormone metabolism (Gazaryan et al., 1996), formation of lignin and liposites, crosslinking of cell walls (Barcelo and Pomar, 2001; Passardi et al., 2004b), cell growth and elongation, and various defense processes against biotic and abiotic stresses (Schopfer et al., 2002; Liszkay et al., 2004; Bindschedler et al., 2006). For example, *Arabidopsis* peroxidases *AtPrx33* and *AtPrx34* are associated with root elongation (Passardi et al., 2006), and *AtPrx72* has an important role in lignification (Herrero et al., 2013). Cotton *GhPOX1* is involved in cotton fiber elongation by means of maintaining high levels of reactive oxygen species production (Mei et al., 2009). The expression of several *ZmPRX* is altered significantly in response to abiotic stress based on microarray analysis in maize, suggesting that these genes play important role in resistance to abiotic stress (Wang et al., 2015). It was also reported that either present or lack of specific peroxidase isoforms appears to be correlated to a particular cellular process or participating a particular protein localization (Loukili et al., 1999; Allison and Schultz, 2004). Genome-wide analysis of this large multigenic family will be greatly helpful to understand its physiological roles and characteristics. In recent years, the PRX family has been widely studied in many species, such as *Arabidopsis thaliana* (Tognolli et al., 2002), rice (Passardi et al., 2004a), and maize (Wang et al., 2015).

Potato (*Solanum tuberosum*) is one of the most important food crops in the world. Potato tubers are rich in nutrients and are valuable processing raw materials for food industry (Pang, 2019). However, potato contains rich phenolic substances, which are the substrates of peroxidase reaction and lead to an enzymatic browning reaction frequently caused by POD (peroxidase) in the storage and potato processing (Zhu and Hu, 2013), affecting the quality of products (Zhou et al., 2010; Zhu, 2017). In addition, potato plants are often subjected to various types of abiotic and biotic stresses during growth and development. Although *PRX* gene family members play important roles in the plant growth and development, their functions in potato are poorly deciphered. Thoroughly analyzing the *PRX* gene family in potato is a primary step to understand its physiological roles and characteristics.

In this study, a systematic investigation of the *PRX* gene family in potato, including *PRX* gene structure, chromosomal localization, gene duplication and phylogenetic relationship, was performed using the sequences from the genome database. Moreover, the tissue-specific expression profile and expression patterns under abiotic stresses such as drought, heat and salt treatments were also investigated. The objectives of this study were to identify and assess the sequence structures of the potato *PRX* gene family and analyze the evolutionary relationship of *PRX* gene family in plants. The results of this study provide a solid foundation for the next phase functional investigation of *PRX* genes in potato.

## Materials and Methods

### Screening and Domain Identification of Potato PRX Proteins

The protein sequences of the *Arabidopsis thaliana* PRX members (Tognolli et al., 2002) were downloaded from the *Arabidopsis* database^1^. These protein sequences of *Arabidopsis* were used as the queries to identify the PRX orthologs in potato using the BLASTP tool SpudDB^2^ and Phytozome v12.1^3^. Proteins with more than 30% similarity to the query sequence and an *E* < E^–10^ were selected. The domains for PRX proteins were further confirmed using the Conserved Domain Database of NCBI^4^. The sequences possessing PRX conserved domain were selected as the final candidates of *PRX* genes (*StPRXs*) and were renamed according to their physical position in the potato genome. The information of these genes was obtained from Phytozome^5^, including gene IDs, physical position, gene sequence and protein sequence. The parameters for the predicted StPRX proteins were calculated using online ExPASy tools (Gasteiger et al., 2003)^6^.

### Gene Structure and Conserved Motif Analysis of Potato PRX Protein

The *StPRX* gene structures were identified using the Gene Structure Display Server (GSDS^7^; Guo et al., 2007). The conserved motifs were identified using the MEME software (version 5.0.3^8^; Bailey et al., 2009). Parameters were set as 20 motifs with the optimum motif width of 50–300 residues. The conserved motifs were then further annotated with the CDD program^9^ (Marchler et al., 2017).

### Phylogenetic Analysis of PRX Proteins

The protein sequences of StPRXs were aligned using the multiple sequence alignment tool ClustalX (Thompson et al., 1997). The phylogenetic tree of PRX family proteins was generated using the MEGA-X maximum-likelihood model (Kumar et al., 2016) with 1000 bootstrap replicates. Orthologs identification method is based on a report (Blanc, 2004). To identify putative orthologs between two different species, each sequence from potato was searched against all sequences from maize and *Arabidopsis* using the BLASTN tool (Altschul et al., 1997). For each query sequence, the best hit among those that met the criteria of alignment ≥ 300 bp and similarity ≥ 40% was considered as the ortholog.

### Chromosomal Location and Gene Duplication

Information of the chromosomal location image of *StPRX* genes was retrieved by the MapInspect tool^10^. To assess gene duplication, the parameters for the proportion of overlap and the similarity between the two sequences were set to be > 70% (Gu et al., 2002; Yang et al., 2008). Two nearby duplicated genes were defined as tandem duplicated genes when the physical space between them was less than 100 kb and contained less than three intervening genes (Wang et al., 2010), while any other two duplicated genes that did not meet the condition of tandem duplicated genes, including those located on the same chromosome or different chromosomes, were all defined as segmental duplicated genes.

### Expression Analysis

The FPKM (fragments per kilobase per million) values of *StRPX* in various tissues and treatments (salt, drought and heat) and their control (CK) generated by RNA-seq (DM_v4.03) were extracted from the Potato Genome Database (see footnote). The expression profile of *StRPX* genes was generated using the R package^11^ of the heatmap function (Warnes et al., 2016).

### Plant Treatments and Quantitative Real-Time PCR Analysis

T virus-free plantlets (*S. tuberosum* L. autotetraploid cultivar Zhongshu 3) were generated by *in vitro* nodal cutting method. Potato shots placed on full MS solid medium were cultured in a growth chamber under the condition of 22°C and 16 h light/8 h dark photoperiod for 1 month. The plantlets were transplanted into a tray with a half-strength modified Hoagland solution (Hammer et al., 1978) for 6 days, and then were exposed to the abiotic stress conditions, including heat (35°C), drought (260 mM mannitol) and salt (150 mM NaCl) treatments. Untreated plantlets were used as control (CK). The treated and control plantlets were collected 6 h after treatment and then stored at −80°C before RNA extraction.

The total RNA of the plantlets was extracted using TRIzol reagent (Invitrogen)^12^ according to the manufacturer’s instructions. The cDNA samples were then assessed by qRT-PCR using SYBR Premix Ex Taq (Takara). *Actin* was used as an internal control gene. Three biological replicates (each containing 6 plants) and three technical replicates were measured for each treatment. The relative expression level of a gene was calculated according to the 2^–ΔΔ*Ct*^ method (Livak and Schmittgen, 2001). The primers used for qRT-PCR analysis are listed in Supplementary Table S1.

## Results

### Identification and Characterization of *PRX* Genes

Using 73 *Arabidopsis* PRX sequences as queries (Tognolli et al., 2002) and validating the candidate sequences by conservative domain analysis based on CDD, a total of 102 PRX genes (*StPRXs*) were identified from potato genome and were renamed from *StPRX1* to *StPRX102* based on their physical position on chromosomes (Table 1). However, the location of *StPRX1* on the chromosome could not yet be defined because it was located in the unmapped scaffold. The protein lengths of the 102 *StPRX* genes varied from 152 (*StPRX29*) to 592 (*StPRX60*) amino acids, with an average of 310.8 amino acids. The molecular weights ranged from 16866.8 Da (*StPRX29*) to 63735.06 Da (*StPRX60*). The theoretical isoelectric points (pI) of these *StPRX* genes varied from 4.43 (*StPRX20*) to 10.02 (*StPRX81*). The detailed information for *StPRX* genes was listed in Table 1.

**TABLE 1:** The characters of 102 *PRX* gene family members in *Solanum tuberosum.*

Gene name	Gene ID	Location (bp)	Chr.	PL (aa)	MW (Da)	PI
*StPRX1*	PGSC0003DMG400011948	22096415..22097488	0	299	32921.88	5.23
*StPRX2*	PGSC0003DMG400032147	1754474..1757591	1	305	33739.61	8.10
*StPRX3*	PGSC0003DMG400032199	1761660..1764244	1	324	34984.22	4.60
*StPRX4*	PGSC0003DMG400016371	3596648..3598780	1	440	48820.52	6.32
*StPRX5*	PGSC0003DMG400014725	6806225..6809227	1	309	34613.11	8.58
*StPRX6*	PGSC0003DMG400014726	6812744..6815906	1	252	27532.73	5.63
*StPRX7*	PGSC0003DMG400014728	6864443..6867718	1	218	24433.11	5.65
*StPRX8*	PGSC0003DMG400024019	45907335..45908930	1	235	25675.13	5.86
*StPRX9*	PGSC0003DMG400011458	47297311..47300055	1	297	32037.04	4.83
*StPRX10*	PGSC0003DMG400022848	59916267..59918783	1	325	35564.60	8.56
*StPRX11*	PGSC0003DMG400022850	59968801..59970227	1	325	35441.54	7.95
*StPRX12*	PGSC0003DMG401018293	78690854..78691752	1	258	28158.11	7.65
*StPRX13*	PGSC0003DMG400012589	81814094..81815527	1	331	35958.90	8.38
*StPRX14*	PGSC0003DMG400025803	84743624..84745033	1	311	34505.20	5.33
*StPRX15*	PGSC0003DMG402015497	20759728..20761348	2	331	36729.25	9.34
*StPRX16*	PGSC0003DMG400025478	23460843..23462610	2	326	35405.94	5.99
*StPRX17*	PGSC0003DMG400022955	32712360..32714253	2	253	27718.73	8.74
*StPRX18*	PGSC0003DMG400010715	32796108..32797987	2	253	27718.73	8.74
*StPRX19*	PGSC0003DMG400022342	35169225..35172205	2	363	38682.57	4.69
*StPRX20*	PGSC0003DMG400022341	35183633..35186491	2	358	38031.64	4.43
*StPRX21*	PGSC0003DMG400016453	36329136..36330486	2	326	37243.99	8.90
*StPRX22*	PGSC0003DMG400013654	37854602..37856626	2	324	36500.79	5.77
*StPRX23*	PGSC0003DMG400003654	39070302..39074823	2	319	34770.84	9.35
*StPRX24*	PGSC0003DMG400012668	41145199..41150765	2	316	35141.91	4.95
*StPRX25*	PGSC0003DMG400024967	43038498..43040517	2	332	36390.55	9.22
*StPRX26*	PGSC0003DMG400010061	44288870..44290076	2	260	28291.97	6.88
*StPRX27*	PGSC0003DMG400010064	44310085..44311431	2	325	35728.53	6.42
*StPRX28*	PGSC0003DMG400000511	46773640..46774626	2	328	36508.42	8.57
*StPRX29*	PGSC0003DMG400020252	48001688..48003039	2	152	16866.80	5.05
*StPRX30*	PGSC0003DMG400005062	2282144..2284899	3	334	36318.98	6.32
*StPRX31*	PGSC0003DMG400022567	3855707..3861892	3	445	49360.67	8.37
*StPRX32*	PGSC0003DMG400022541	3883852..3886414	3	333	36913.24	8.47
*StPRX33*	PGSC0003DMG400014867	7132984..7135756	3	331	35845.02	8.53
*StPRX34*	PGSC0003DMG400024253	41651231..41652456	3	317	34448.21	6.98
*StPRX35*	PGSC0003DMG400015801	43497865..43500054	3	319	34444.9	8.87
*StPRX36*	PGSC0003DMG400000559	46475042..46476231	3	290	31249.31	5.83
*StPRX37*	PGSC0003DMG401002540	60230456..60232244	3	355	38956.21	5.28
*StPRX38*	PGSC0003DMG400024813	58199228..58201394	4	273	29849.00	5.59
*StPRX39*	PGSC0003DMG400025084	61089768..61091391	4	264	28737.56	7.61
*StPRX40*	PGSC0003DMG401025083	61081697..61083354	4	348	38241.36	5.88
*StPRX41*	PGSC0003DMG402025083	61099781..61102899	4	359	39717.90	7.58
*StPRX42*	PGSC0003DMG400006386	65556167..65558314	4	323	35728.04	8.77
*StPRX43*	PGSC0003DMG400005152	69721596..69723452	4	310	34424.07	5.71
*StPRX44*	PGSC0003DMG400003748	70184517..70186504	4	331	36595.03	8.29
*StPRX45*	PGSC0003DMG400009950	71149878..71152497	4	271	29647.46	8.64
*StPRX46*	PGSC0003DMG400015584	7054308..7055315	5	335	37292.16	8.03
*StPRX47*	PGSC0003DMG400018624	10018838..10020432	5	485	52645.89	5.18
*StPRX48*	PGSC0003DMG400020975	23721320..23722830	5	241	26599.03	8.44
*StPRX49*	PGSC0003DMG400005272	42500860..42503146	5	322	35270.83	8.77
*StPRX50*	PGSC0003DMG400005279	42524175..42525868	5	327	35795.19	8.26
*StPRX51*	PGSC0003DMG400005273	42534489..42535934	5	327	35736.75	7.54
*StPRX52*	PGSC0003DMG400015035	42586204..42587629	5	327	35795.79	7.57
*StPRX53*	PGSC0003DMG400006993	45422092..45429842	5	273	29118.81	5.92
*StPRX54*	PGSC0003DMG400023491	50994131..50995456	5	334	36697.30	9.09
*StPRX55*	PGSC0003DMG400014055	31519344..31521327	6	319	34526.11	9.41
*StPRX56*	PGSC0003DMG400030764	40825442..40828587	6	312	34193.31	8.62
*StPRX57*	PGSC0003DMG400030430	56939983..56941180	6	316	35275.13	5.73
*StPRX58*	PGSC0003DMG400030382	57556039..57558160	6	312	33614.90	6.06
*StPRX59*	PGSC0003DMG400004090	10927329..10929258	7	329	36314.51	9.33
*StPRX60*	PGSC0003DMG400000694	44979030..44982593	7	592	63735.06	8.90
*StPRX61*	PGSC0003DMG400000693	44995516..44996807	7	331	35726.00	9.03
*StPRX62*	PGSC0003DMG400025492	47159208..47160591	7	328	36021.11	7.51
*StPRX63*	PGSC0003DMG400025491	47171967..47173483	7	329	35706.95	8.97
*StPRX64*	PGSC0003DMG400025490	47178254..47179727	7	329	35804.15	8.96
*StPRX65*	PGSC0003DMG400020437	50315313..50316847	7	224	23723.53	8.30
*StPRX66*	PGSC0003DMG400020494	1070500..1071459	8	319	35247.52	6.35
*StPRX67*	PGSC0003DMG400005872	5883543..5889021	8	345	38898.58	8.71
*StPRX68*	PGSC0003DMG400029546	44803067..44804385	8	336	37119.67	9.41
*StPRX69*	PGSC0003DMG400001774	6294037..6297308	9	324	36832.21	7.12
*StPRX70*	PGSC0003DMG400026575	13125916..13128279	9	316	33238.39	8.63
*StPRX71*	PGSC0003DMG400037550	13143159..13144652	9	204	22597.61	8.70
*StPRX72*	PGSC0003DMG400024285	50464266..50465966	9	322	35077.57	8.88
*StPRX73*	PGSC0003DMG400035475	28321075..28323202	10	322	35248.38	6.39
*StPRX74*	PGSC0003DMG400006679	49022687..49023709	10	254	28268.72	5.24
*StPRX75*	PGSC0003DMG400020800	49084193..49085040	10	250	27069.43	5.68
*StPRX76*	PGSC0003DMG400020799	49151590..49152437	10	250	27197.52	5.68
*StPRX77*	PGSC0003DMG400020798	49172630..49173264	10	179	18985.45	4.83
*StPRX78*	PGSC0003DMG400020801	49258779..49259876	10	300	32593.1	9.03
*StPRX79*	PGSC0003DMG400010465	54852020..54854941	10	329	37248.41	6.63
*StPRX80*	PGSC0003DMG401010480	54880317..54881406	10	336	36523.78	9.27
*StPRX81*	PGSC0003DMG400010479	54899402..54900633	10	383	42139.81	10.02
*StPRX82*	PGSC0003DMG400034594	58853562..58854760	10	203	21774.81	6.07
*StPRX83*	PGSC0003DMG400016223	5090584..5091685	11	337	37722.07	5.46
*StPRX84*	PGSC0003DMG400019492	5101081..5102175	11	335	36560.76	8.51
*StPRX85*	PGSC0003DMG400019491	5104999..5106087	11	335	36652.79	8.32
*StPRX86*	PGSC0003DMG400019490	5107998..5108778	11	332	36196.38	8.51
*StPRX87*	PGSC0003DMG400019474	5116202..5117294	11	232	25085.99	8.56
*StPRX88*	PGSC0003DMG400019473	11796060..11796799	11	305	33236.00	8.67
*StPRX89*	PGSC0003DMG400018031	11937469..11938723	11	178	19158.80	8.26
*StPRX90*	PGSC0003DMG400015106	12404341..12405565	11	319	34436.96	6.52
*StPRX91*	PGSC0003DMG400027614	20684710..20686486	11	300	32441.69	6.94
*StPRX92*	PGSC0003DMG400019766	2724753..2727047	11	252	26855.16	4.88
*StPRX93*	PGSC0003DMG400015548	45091925..45093213	11	326	34682.53	4.84
*StPRX94*	PGSC0003DMG400039106	2392106..2393703	12	328	36283.93	9.22
*StPRX95*	PGSC0003DMG400024329	6654188..6656445	12	324	35318.86	9.00
*StPRX96*	PGSC0003DMG402024332	6656986..6658371	12	335	37699.31	8.05
*StPRX97*	PGSC0003DMG401029332	6660893..6662191	12	322	35245.52	8.65
*StPRX98*	PGSC0003DMG400024330	6664412..6668643	12	322	35330.71	8.77
*StPRX99*	PGSC0003DMG400021801	7978370..7980568	12	328	36224.44	8.66
*StPRX100*	PGSC0003DMG400020388	11649782..11651610	12	353	38921.57	8.79
*StPRX101*	PGSC0003DMT400075415	57162212..57162790	12	154	17167.35	9.47
*StPRX102*	PGSC0003DMG402029332	57165238..57166864	12	318	34969.76	8.81

**Chr., chromosome; PL, protein length; MW, molecular weight; PI, theoretical isoelectric points.**

### Phylogenetic Analysis

**FIGURE 1:**
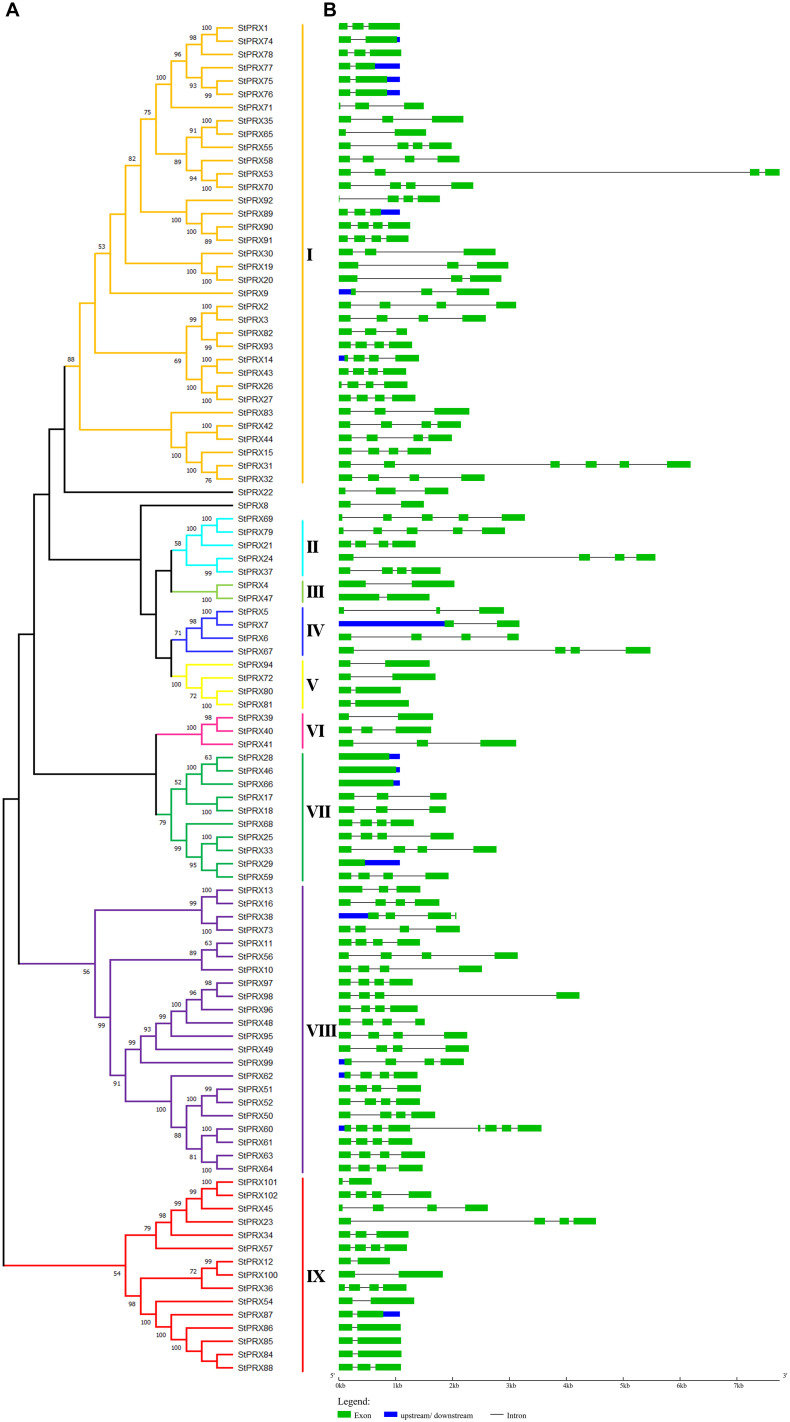
Phylogenetic tree and gene structure of *StPRX* genes. **(A)** The maximum-likelihood phylogenetic tree generated by MEGA X with bootstrapping analysis (1,000 replicates). Each subfamily is distinguished by different colors. **(B)** The exon-intron structure of *StPRX* genes generated by the online software GSDS. The horizontal black lines and the green boxes represent introns and exons, respectively. The blue boxes represent upstream or downstream sequences. The sizes of exons and introns can be estimated according to the scale at bottom.

To reveal the evolutionary relationship of the *PRX* gene family, an unrooted phylogenetic tree (Figure 1A) was obtained basing the MEGA-X maximum-likelihood model. The 102 *StPRXs* were classified into 9 subfamily (I-IX) with the bootstrap values (≥50%) on the phylogenetic tree. However, 2 *StPRX* genes (*StPRX 8* and *StPRX22*) could not be assigned to any of the 9 subfamilies due to the low bootstrap values (<50%). Among these 9 groups, subfamily I possessed the largest clade, which contained 35 *StPRX* genes, followed by group VIII, which had 22 PRX members. These two subfamilies accounted for 55.88% of the total *StPRXs.* In contrast, groups III and IV only had two or three *StPRX* genes.

**FIGURE 2:**
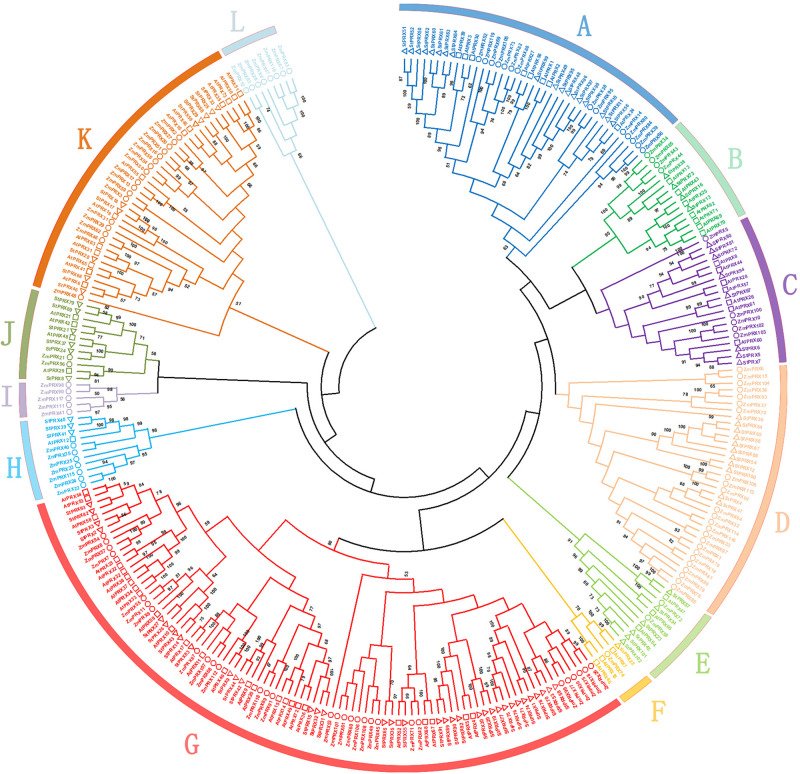
Phylogenetic tree of potato, maize and *Arabidopsis* PRXs. Each PRX subfamily is shown by a specific color. The phylogenetic tree was constructed by MEGA X with 1000 bootstrap replicates. The circles, squares, and triangles represent maize, *Arabidopsis* and potato PRX proteins, respectively.

To further explore the evolutionary process of the PRX family in potato, the 102 PRX protein sequences of potato were aligned with 73 PRX proteins of *Arabidopsis thaliana* and 119 PRX proteins of maize (Tognolli et al., 2002; Wang et al., 2015). The phylogenetic tree was divided into 12 different groups (groups A–L; Figure 2). Among these groups, group G was the largest, which contained 92 PRX members, including 35 of potato, 30 of maize and 27 of *Arabidopsis*. Groups A, D and K also had a large number of genes, containing 40, 35, and 40 members, respectively. In contrast, group F was the smallest, which contained only 5 members, including 1 of potato, 1 of maize and 3 of *Arabidopsis*. Interestingly, although group D was large, it had no members from *Arabidopsis*. Moreover, group I and L only contained 5 and 8 members from maize, respectively. A few groups were supported by low bootstrap values, which might be due to the relative less informative character positions beside the conserved PRX domains. This scenario has been also found in the analysis of other gene families (Li et al., 2006; Wang et al., 2018). In addition, a total of 82 ortholog pairs were identified between potato and *Arabidopsis* (St-At; Supplementary Table S4). However, only four ortholog pairs were retrieved between potato and maize (St-Zm; Supplementary Table S5). The orthologs between potato and *Arabidopsis* were much greater than that between potato and maize, probably because of the closer evolutionary distance between these eudicot species.

### Gene Structure and Protein Motif Analysis of *StPRX*

Structure analysis of *StPRX* genes showed that the number of introns varied from 0 to 7 (Figure 1B). Most of them had 1–3 introns, with *StPRX60* containing the maximum (7) while four genes lacked introns (*StPRX28*, *StPRX29*, *StPRX46*, and *StPRX66*) (Figure 1B). Genes with similar exon/intron structure were grouped together, but structural variation was also found among these *StPRX* genes (Figure 1).

**FIGURE 3:**
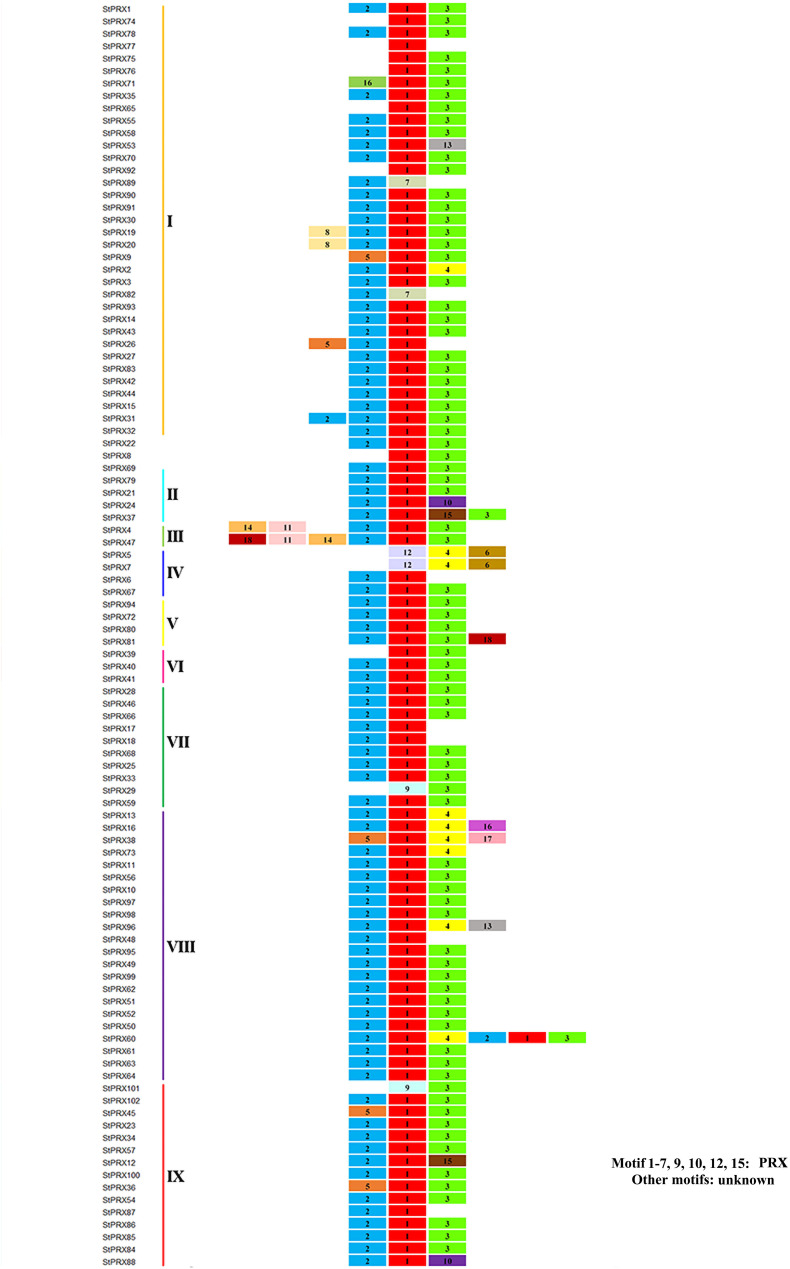
Conserved motifs of PRX proteins in potato. The protein list on the left is the same as that in Figure 1. The genes of different subfamilies are highlighted with different colors. Different motifs are exhibited with different colored boxes and numbers (1–18). The detailed sequences of motifs are listed in Supplementary Table S2.

To investigate the diversity of motif components among *StPRXs*, the motif distribution in 102 StPRX proteins was investigated using the online tool MEME program. A total of 18 conserved motifs were identified (Figure 3 and Supplementary Table S2). The majority of StPRX proteins contained two to three conserved motifs. The StPRX proteins on the same branch had similar conserved motif composition and sorting order, suggesting that StPRX proteins in the same branch might share similar function. Using the CDD tool, a total of 11 motifs (motif 1/2/3/4/5/6/7/9/10/12/15) were functionally annotated for the components of the conserved PRX domain (Figure 3 and Supplementary Table S2). All the members of the potato PRX family contained at least one motif belonging to the typical domains of PRX family. In addition, some motifs appeared to be unknown in function. For example, the functions of three motifs (motif 11/14/18) in subgroup III and of four motifs (motif 8/13/16/17) in subgroups I, III, V, and VIII were yet to be determined.

### Chromosomal Locations and Duplications of *StPRX* Genes

**FIGURE 4:**
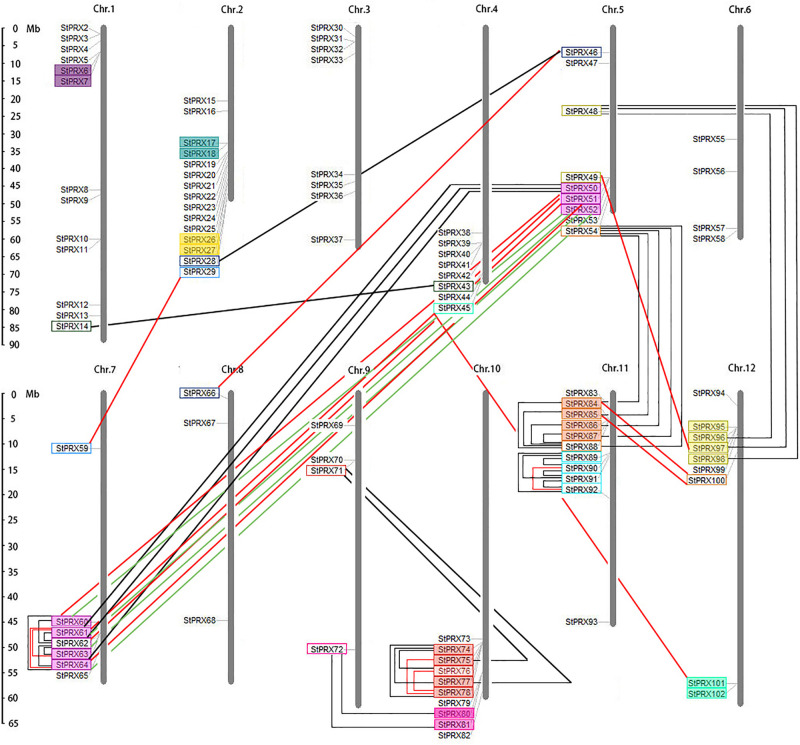
Chromosomal locations of potato PRX genes. The scale on the left presents the length of potato chromosomes (Mb). Tandem duplicated gene pairs are displayed with boxes of the same color. Segmented duplicated gene pairs are displayed with boxes connected by lines.

To reveal the genome organization and distribution of *StPRX* on different chromosomes in potato, a graph of chromosomes was constructed using the MapInspect tool. A total of 101 out of the 102 *StPRX* genes were located on the 12 potato chromosomes (Figure 4). Among them, the largest number of *StPRX* genes (15) was located on chromosome 2, followed by chromosomes 1 (13), and chromosomes 11 (11) and 10 (10). In contrast, only a few *StPRX* genes were located on chromosomes 8 (3), 6 (4), and 9 (4). In addition, some chromosomes showed a dense cluster of *StPRXs*, such as near the telomeric region of chromosomes 2, 4, 5, 7, 10, 11, and 12. Gene duplication events, including segmental duplication and tandem duplication, are important for the expansion of the gene family during the process of the evolution (Cannon et al., 2004). In this study, a total of 83 *StPRX* gene pairs were identified from the phylogenetic and comparative analysis (Figure 4), among which 57 pairs were found to be involved in the segmental duplication events, and 26 pairs were confirmed to be tandem duplicated genes (Figure 4 and Supplementary Table S3). The number of segmental duplication gene pairs was twice as many as that of the tandem duplicated, and most of the tandem duplicated gene pairs were densely distributed at the end of chromosomes 5, 7, 10, 11, and 12.

### Expression Patterns of *StPRX* Genes

**FIGURE 5:**
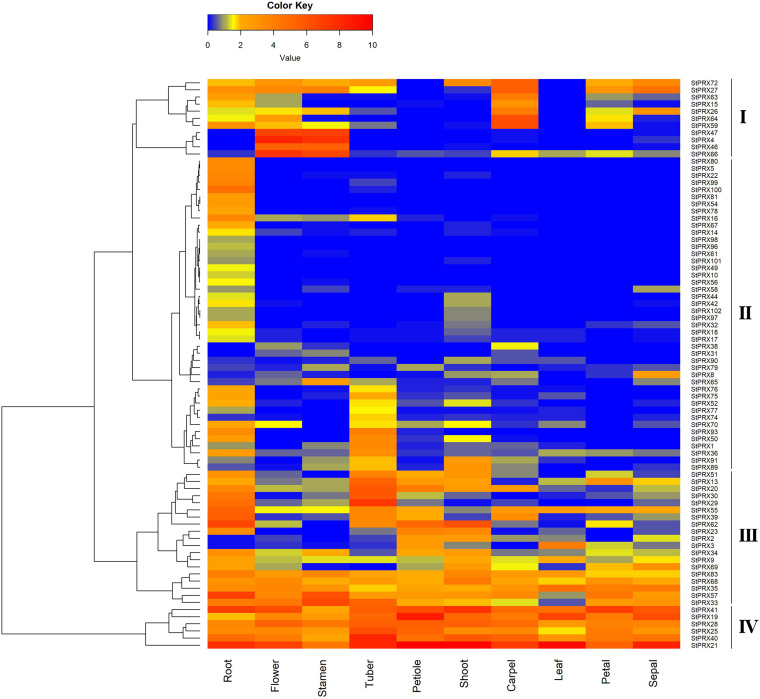
Expression profiles of *StPRX* genes in different tissues. FPKM values for *StPRX* genes were transformed by log_2_(FPKM+1). The heatmap was generated using the R package of the heatmap function.

To further explore the expression patterns of the *StPRX* genes, the transcript data of major tissues was obtained from the public genome database, including root, shoot, petal, carpel, sepal, stamen, tuber, leaf, flower and petiole. A heatmap was generated based on the transcript data of 80 *StPRX* genes, and the other 22 genes were excluded from the heat map analysis due to the low expression level (FPKM < 0.5) or lack of expression in all tissues (Figure 5). As shown in Figure 5, some *AtPRX* genes exhibited distinct tissue-specific expression patterns, while others were active in the whole plant. The 80 *StPRX* genes were grouped into four groups (Figure 5). Six genes (*StPRX41*/*19*/*28*/*2*5/*40*/*21*) were included in group IV, which had especially abundant expression level in all of the developmental stages, suggesting that these genes might play important basic roles in all development stages of plant. A total of 19 genes (*StPRX51*/*13*/*20*/*30*/*29*/*55*/*39*/*62*/*23*/*2*/*3*/*34*/*9*/*69*/*83*/*68*/*35*/*57*/*33*) were included in group III, which had high expression levels in most of the analyzed tissues. In contrast, 44 genes in group II exhibited low expression or no expression in the most of the tissues analyzed. However, most of them showed relatively high expression in root than in other tissues. This was also observed in the *PRX* family of maize (Wang et al., 2015) and *Arabidopsis* (Tognolli et al., 2002). Therefore, the expression patterns of *StPRX* genes may reflect the correlation with their functions.

### Expression of *StPRX* Genes in Response to Abiotic Stress

**FIGURE 6:**
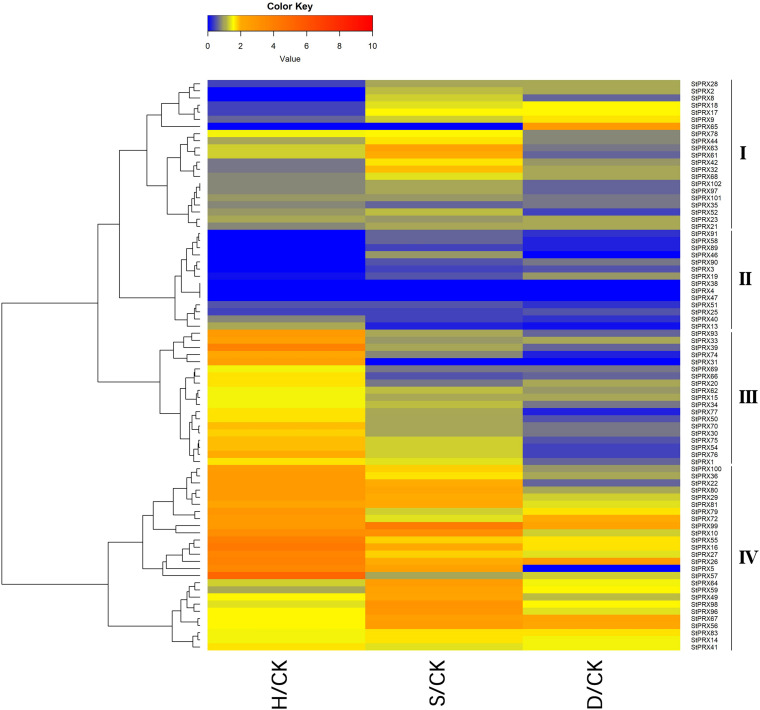
Expression changes of StPRX genes under heat (H), salt (S) and drought (D) stresses. The expression change is indicated by the ratio of FPKM value of the treatment to that of the control (CK). The heatmap was generated using the R package of the heatmap function.

To further investigate the response of the *StPRX* genes subjected to different stresses, including heat, salt and drought, the relative expression levels among the tissues were measured based on the expression FPKM values (stress/control) (Figure 6). The expression levels of different genes showed great variation under the various types of treatments. Under heat stress, most of the *StPRX* genes in groups III and IV were significant upregulated, such as *StPRX93/33/39/74/31/*100/36/22, whereas most of the *StPRX* genes in groups I and II exhibited significant downregulation, such as *StPRX 28*, *-18* and *-17.* Most of the *StPRX* genes showed downregulation under the stress of drought, in contrast to the performance under heat stress, and some *StPRX* genes in groups II and III were extremely sensitive to the drought stress and exhibited significant downregulation, such as *StPRX4/13/31/38/46/57/51/58/74/77/89/91*. In terms of saline stress, the genes exhibited diverse responses, the genes in group IV were significantly upregulated, and the genes in group II showed significant downregulation, while the genes in groups I and III only showed slight changes, implying the functional dissimilation among the *StPRX* genes.

**FIGURE 7:**
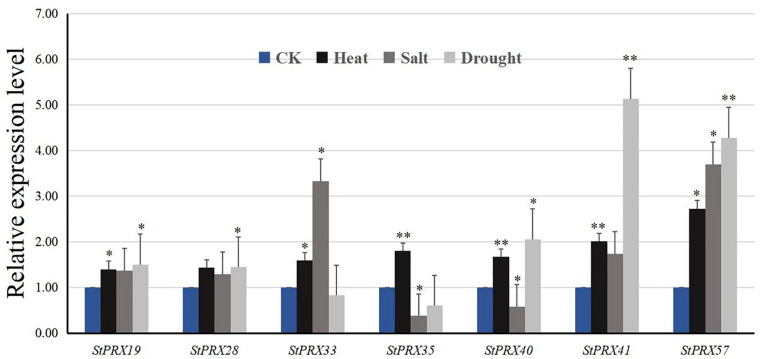
Relative expression levels of 7 *StPRX* genes in response to heat, salt and drought stresses after 6 h treatment compared with that of control (CK). Error bars are standard deviations of three biological replicates. *P < 0.05, **P < 0.01.

Seven *StPRX* genes (*StPRX19/28/33/35/40/41*/*57*) with high expression levels in all organs were selected for further qRT-PCR analysis under different abiotic stresses. The 7 genes showed different levels of response to the three abiotic stress treatments (Figure 7). All of the selected genes were up-regulated under heat stress, and the expression change of *StPRX57* was over twofold. For saline stress, most of the selected genes were up-regulated, and the upregulation of *StPRX33 and StPRX57* was more than threefold. In response to drought stress, 5 genes (*StPRX19/28/40/41*/*57*) were upregulated, whereas *StPRX33 and StPRX35* appeared to be slightly down-regulated. Notably, 2 genes (*StPRX41 and StPRX57*) were extremely sensitive to drought stress. Their expression levels increased 4 folds compared to that of control.

## Discussion

PRXs are plant-specific enzymes that have multiple functions in the growth and development of plants. It has been confirmed that *PRX* genes are widely involved in stress response in many species (Gray and Montgomery, 2003; Xue et al., 2008). To date, the comprehensive genome-wide analysis of the PRX gene family has been performed in many plant species, including *Arabidopsis* (Tognolli et al., 2002), rice (Passardi et al., 2004a), and maize (Wang et al., 2015). In this study, a total of 102 *StPRX* genes were identified. The number was more than that in *Arabidopsis* (containing 73 *PRX* genes), but slightly less than that of maize (119) and rice (138). According to the phylogenetic tree of the 288 PRX family members from *Arabidopsis*, maize and potato, we found that some groups only contained members from one or two species. In addition, orthologs between potato and *Arabidopsis* (82 pairs) were much more than those between potato and maize (4 pairs), suggesting that the *PRX* gene family underwent a specific expansion after the three species diverged each other in the evolutionary path of speciation, especially after the divergence of monocots and eudicots.

We have seen that the StPRX proteins showing similar domain architectures and motif constitutions were usually grouped in the same subfamily (Figure 3), and the structural constitutions of the *StPRX* genes in each group were basically in consistence with the result of phylogenetic analysis (Figure 1). Similar phenomenon have also been found in many other species, such as maize (Wang et al., 2015) and rice (Passardi et al., 2004a). As protein function is mostly determined by its domain structure, these results imply that the StPRX proteins with similar domain architectures and motif constitutions could probably perform similar functions.

It is known that the structural diversity of genes drives the evolution of multigene families. The intron/exon structure variation is a cause of gene diversity. Many studies have shown that introns are specifically inserted into and retained in the genome during evolution (Rogozin et al., 2003). The loss and insertion of new introns appear to be frequent events, which may result in diverse functional consequences in gene evolution. The duplication from an ancient gene formed by shuffling of small exons could be the reason that resulted in the genes with a relatively high number of introns (Gilbert et al., 1997). In maize, it is speculated that the intron variation among *PRX* genes might result from the depletion and duplication of single introns in the course of evolution (Wang et al., 2015). In this study, we found that the number of introns in *StPRX* genes was also quite variable (varying from 0 to 7), and the proteins in the same subfamily were not completely identical in terms of their intron/exon structure and motifs (Figures 1, 3), implying that exon shuffling might be a main pattern of *StPRX* evolution, which might be the main contributors to the functional diversity of the potato PRX family. Among the 102 *StPRX* genes, more than half of them consist of 3 introns and 4 exons. This 3-intron/4-exon model is also represented a significant proportion in *Arabidopsis* (Tognolli et al., 2002) and in rice (Passardi et al., 2004a), suggesting that it is an ancestral intronic model of *PRX* genes.

Gene duplication events, including segmental and tandem duplication are important for the expansion of the gene family during the process of the evolution (Cannon et al., 2004). Gene duplication events can theoretically produce two gene copies, and one or both copies can acquire the novel gene functions for adaptation under a smaller selective pressure of evolution (Van de Peer et al., 2009). Each paralog is specialized for a specific functional assignment (Zhang, 2003), which often leads to the expansion of gene family (Cannon et al., 2004). The segment duplication event refers to the duplication of large fragments of the genome, which may have derived from segmental, chromosomal or whole genome duplications with many losses and rearrangements (Zhang, 2003). Tandem duplication affects a limited number of genes (one or more neighboring genes); it often derives from unequal crossing-over (Achaz et al., 2000) and multiple episodes of unequal crossovers. In addition, the retrotransposition event of cDNA also contributes to the expansion of gene family, which is characterized by the loss of all introns and related regulatory sequences and by a random insertion within the genome. In our study, a total of 83 duplicated gene pairs were identified in potato *PRX* gene family, including 57 segmental duplication gene pairs and 26 tandem duplicated genes pairs (Supplementary Table S3), which were much more than those in maize (28 duplicated gene pairs; Wang et al., 2015) and rice (Passardi et al., 2004a). The segmental duplication gene pairs were twice as many as the tandem duplicated gene pairs, indicating that segmental duplication might play the dominant role in the expansion of the potato PRX family. Most of the tandem duplicated gene pairs were densely distributed in telomeric regions of chromosomes (such as on chromosomes 5 and 7), and many tandem duplicated genes shared high similarity with the same segmental duplication genes, implying that most of the tandem duplicated genes might appear after the segmental duplication events. Notably, two gene pairs (*StPRX28/46, StPRX46/66*) met the criteria of segmental duplication gene pairs but had no introns, suggesting that they might likely be generated by retrotransposition. Strictly speaking, therefore, they might not be segmental duplication gene pairs.

Gene expression pattern is an important aspect related to gene function. In this study, among the 102 *StPRX* genes, except for 22 with weak or without expression in all of the tissues examined, the rest all exhibited distinct patterns of tissue-specific expression (Figure 5) and response to stress (Figure 6), indicating the functional dissimilation of StPRX proteins. This is consistent with the results of phylogenetic and protein motif analyses. Several genes in group IV were expressed in all organs (Figure 5), suggesting that they might play basic roles for the plant. Notably, the largest number of *StPRX* genes with high expression levels was found in root (Figure 5). Similar observations were also reported in maize (Wang et al., 2015), *Arabidopsis* (Tognolli et al., 2002), and rice (Passardi et al., 2004a), suggesting that the *PRX* family might be critical for root function in plants. In maize, there are many cell wall or membrane-bound PRXs in root (Mika et al., 2008; Šukaloviæ et al., 2015); several *ZmPRX* genes from roots are regulated by methyl jasmonate, salicylic acid and pathogen elicitors (Mika et al., 2010); and some genes (*ZmPRX26*/*42*/*71*/*75*/*78*) highly expressed in root show significant responses to H_2_O_2_, SA, NaCl, and PEG treatments (Wang et al., 2015). In *Arabidopsis*, two *AtPRX* genes (*AtPrx33*/*34*) are associated with root elongation (Passardi et al., 2006). Interestingly, the five *ZmPRX* genes (*ZmPRX26*/*42*/*71/75*/*78*) and two *AtPRX* genes (*AtPrx33*/*34*) were all clustered in grouped G in this study (Figure 2). Most *StPRX* genes in group G (Figure 2) were included in the subfamily I (Figures 1, 3) with similar gene structure and motif components. The results imply that the *StPRX* genes clustered in group G might also function in root similar to their counterparts in maize and Arabidopsis. Therefore, the results of our study may provide a basis for the functional exploration of the potato *PRX* gene family members.

To analyze the trend of the gene expression derived from qRT-PCR (Figure 7) and the FPKM values, we compared the results from these two different platforms (Figure 6). Overall, similar propensity of gene expression was found between the two different approaches. However, the results of qRT-PCR did not totally agree with the pattern of the gene expression from RNA-seq data. There could be several reasons for the discrepancy. First, the genotypes of potato varieties used in the two experiments were different. A doubled monoploid potato variety (DM) was used in RNA-seq (Xu et al., 2011), whereas an autotetraploid cultivar Zhongshu 3 was used for qRT-PCR analysis. Second, the experimental treatments were different. The plantlets for qRT-PCR were grown under a photoperiod of 16 h light/8 h dark environment, while the materials for RNA-seq were grown in the dark (Xu et al., 2011).

## Conclusion

In this study, a genome-wide investigation and comprehensive analysis of the *PRX* gene family in potato was conducted. The structural diversity of *StPRXs* may reflect their functional diversity. The analysis of expression patterns of *StPRX* genes showed that these genes were expressed distinctly in different tissues of potato, and some might be linked to stress responses. It is important to thoroughly investigate the biological functions of *StPRX* genes, especially the roles in the resistance to abiotic stresses. Our results provide the vital information for the exploration of the functional aspect of the gene family.

## Data Availability Statement

The original contributions presented in the study are included in the article/Supplementary Material, further inquiries can be directed to the corresponding author/s.

## Author Contributions

XY and JYa performed the experiments and analyzed the data. WL collected plant materials and performed the experiments. MQ participated in handling figures and tables. XX designed this research and wrote the manuscript. WW and JYu helped to draft the manuscript. All authors read and approved the manuscript.

## Conflict of Interest

The authors declare that the research was conducted in the absence of any commercial or financial relationships that could be construed as a potential conflict of interest.

## Footnotes

1 http://www.arabidopsis.org/

2 http://solanaceae.plantbiology.msu.edu/

3 https://phytozome.jgi.doe.gov

4 https://www.ncbi.nlm.nih.gov/cdd/

5 https://phytozome.jgi.doe.gov/pz/portal.html

6 http://web.expasy.org/protparam/

7 http://gsds.cbi.pku.edu.cn/

8 http://alternate.meme-suite.org/tools/meme

9 https://www.ncbi.nlm.nih.gov/

10 http://www.plantbreeding.wur.nl/uk/software_mapinspect.html

11 http://www.r-project.org/

12 http://www.invitrogen.com
