# Supplementary material for: Genome-Wide Identification and Expression Analysis of the Class III Peroxidase Gene Family in Potato (Solanum tuberosum L.)
Source: Front Genet. 2020 Dec 3;11:593577. doi: 10.3389/fgene.2020.593577 (PMC7744636; doi:10.3389/fgene.2020.593577)
Supplement: Supplementary file 1 [file Table_1.DOCX]

| **Table S1** Potato *PRX* gene-specific primers used for qRT-PCR analysis. | | |
| --- | --- | --- |
| Primer Name | Forward Primer (5′→3′) Sequence | Reverse Primer (5′→3′) Sequence |
| StPH19 | AGACAGCAACTAACTCCGGA | GCAGAATACCTCCATCACAACC |
| StPH28 | ACGTGACGCTGAAATCAACC | CCTCCGGTTTGAACAACGAG |
| StPH33 | TGCCCAAACTTGTCCTAATGTT | TGCTGTGTTTCCTGACGTTG |
| StPH35 | GCCGTAATCTGCAGACAATTGT | TGGGTTTGCATTCTTTTCTCCT |
| StPH40 | GCCCAACTACGATGTACCCT | TGATACCGATAGTGTGGGCC |
| StPH41 | ACTCCATTTGTCCCAATGCTG | TCACTTGGTCCACTGGTTGA |
| StPH57 | GCTGATCCTACTCTTGCTGC | CAGGGCATTGGATTTCGAGT |
| StActin | GCTTCCCGATGGTCAAGTCA | GGATTCCAGCTGCTTCCATTC |
