# Supplementary material for: Genome-Wide Identification and Expression Analysis of the Class III Peroxidase Gene Family in Potato (Solanum tuberosum L.)
Source: Front Genet. 2020 Dec 3;11:593577. doi: 10.3389/fgene.2020.593577 (PMC7744636; doi:10.3389/fgene.2020.593577)
Supplement: Supplementary file 2 [file Table_2.DOCX]

**Table S2** Sequences of 18 predicted motifs of StPRX proteins

| **Motif** | **Width** | **Motif Sequence** | **Annotation** |
| --- | --- | --- | --- |
| 1 | 99 | EAECPGVVSCADILALAARDSVVLLGGPYWEVPLGRRDGRTSSASEANTNJPSPFSNLSTLJTSFASKGLDLKDLVALSGAHTIGFAHCSSFSNRLYN | secretory_peroxidase super family |
| 2 | 70 | QLKVGFYDKTCPNAESIVKSVVNKAISKDPRLAAALLRLHFHDCFVRGCDASVLLDSTSSNKSEKEADPN | secretory_peroxidase super family |
| 3 | 75 | TPTFFDNYYYKNLKNKKGLLSSDQLLTSDGTTAGJVKLYASNPSTFFKDFAKSMIKMGNIGVLTGNNGEIRKNCR | secretory_peroxidase super family |
| 4 | 69 | GGDDPSIBPKFLPELKKKCPKNGDVNTRVPLDKGSPSKFDNSYFQNIKNGNGILZSDARLYTDATTKDV | secretory_peroxidase super family |
| 5 | 50 | VPAALLRLHFHDCFVRGCDGSVLJDDTKTNSGEKDAPPNLNSLRGYEVID | secretory_peroxidase super family |
| 6 | 50 | DIVKDPDFYAKFGEAMVKLGRVEVLIDGQGEVRKSCRVVNKKPFFFGGFN | secretory_peroxidase super family |
| 7 | 69 | VRGFGIIEDIKREVEKCCPGVVSCADILALAAEDAVSLVGGPSWNVKLGRRDSRRANQGGAEIDJPGPF | secretory_peroxidase super family |
| 8 | 50 | MGFRLSHLSLVLSFVALALAGVAIYRNTYEAIIMNNGSPDFDFLPNGDVL | — |
| 9 | 61 | NFAQRGLSQDDLIALSGGHTLGFSHCDRFQNRJHKFDKRNNVDPSLDPEFAKELKDICPQN | secretory_peroxidase super family |
| 10 | 50 | DNRYYKALKRGKGLLFADQQLMANPKTAKAVNDNARDGGIWFRKEFAAAI | secretory_peroxidase super family |
| 11 | 50 | NFKEKKVKEAGFERFKFEHDLWGGGRHGDKNVFPEEDVDPITPEPEDPEP | — |
| 12 | 65 | CFEFEKGKCGGKWVEVEVGDRDGEWHLANREKDPAAAJLLSDSIKPFGKKNLSPIDMVYLLGGIY | secretory_peroxidase super family |
| 13 | 50 | NCFKDGWDNDGENGDNWSPYWEDRGNYKNLCJQKGRKNFDZHFFLZDFQH | — |
| 14 | 57 | NNRPEGLPGDSQGPDVAIGGGGNNIVQNNVDDQAIPEQNGEARNERSPEEKKENRKN | — |
| 15 | 50 | FNGTNPQDPTMDPEFAQFLRKKCNRNQISDPIPILKLJLPIGWIISSJSP | secretory_peroxidase super family |
| 16 | 50 | CDAAGLJDGLLGJDFEKNFFQNNNKARGFEVKDGIDGEIDKVCGRFNVYC | — |
| 17 | 50 | RDSVLEICEFINPNPSLFGNDFGRIMNELGRJEVKFGKQGZIRCPGDGGD | — |
| 18 | 50 | RVAKARJFKGLRLJCJLLPKLARVFLDHLPEVGRVNEFDNNDVPRVLLLL | — |

–Means no annotation was found
